# Frequency of Right Ventricular Infarction in Inferior Wall Myocardial Infarction

**DOI:** 10.7759/cureus.8238

**Published:** 2020-05-22

**Authors:** Hassam Ali, Shiza Sarfraz, Muhammad Fawad, Zarafshan Shafique

**Affiliations:** 1 Internal Medicine, East Carolina University, Vidant Medical Center, Greenville, USA; 2 Anesthesiology, Bahawal Victoria Hospital, Quaid-E-Azam Medical College, Bahawalpur, PAK; 3 Cardiology, Bahawal Victoria Hospital, Quaid-E-Azam Medical College, Bahawalpur, PAK; 4 Internal Medicine, Bahawal Victoria Hospital, Quaid-E-Azam Medical College, Bahawalpur, PAK

**Keywords:** right heart failure, right ventricular infarction, inferior wall myocardial infarction, iwmi, rvi, right coronary artery (rca), rca, heart attack, myocardial infarction, mi

## Abstract

Background

Ischemic heart disease, particularly inferior wall myocardial infarction (IWMI), is a significant issue in cardiac health. It can further add up to the morbidity and mortality when it is associated with right ventricular infarction (RVI). Elevated ST-segment elevations in the right chest lead number, three (V3R), and four (V4R), can be used to diagnose right ventricular infarction (RVI). The odds of RVI can be identified according to age groups, gender, and risk factors, including diabetes, hypertension, and smoking. This can help in the prevention of right ventricular infarct and its complications by controlling the risk factors which affect the outcome the most.

Methods

A sample of 1000 patients (n=1000) with acute IWMI was evaluated for the incidence of accompanying right ventricular infarct. These patients were then assessed for various known risk factors of myocardial infarction.

Results

Comparing the incidence of RVI against various risk factors, we found that there is an increased incidence of RVI in patients with risk factors that include hypertension and smoking.

Conclusions

The study suggests that IWMI can be accompanied by RVI in almost one-third of the cases, (36% in our study). The odds of RVI are highest in patients of hypertension, and the timely control of certain risk factors will result in reduced incidence and hence the complication associated with right ventricular infarction.

## Introduction

Ischemic heart disease (IHD) is a well-reported entity having a great impact on morbidity and mortality of cardiac patients. Anterior wall myocardial infarction (AWMI) has a destructive course, but the inferior wall myocardial infarction (IWMI) can also be risky, especially if it involves the right ventricle. Right ventricular infarction (RVI) can severely interfere with the hemodynamics, conduction, and valvular irregularities, which can result in sudden death [1]. The objective of this study was to determine the frequency of RVI in cases with acute IWMI. Electrocardiographic evidence was the main criteria for the diagnoses of RVI in all patients of IWMI. A standard 12-lead electrocardiogram (ECG) with right precordial (chest) leads V1R to V6R was performed in all patients. All electrocardiograms were standardized to 10-mm/mV voltage speed and 25-mm/sec paper speed. The diagnosis of RVI was made according to ECG findings. There was a minimum of 1-mm ST elevation in V4R and V3R. ST-segment elevation in lead V4R is the single most powerful predictor of right ventricular involvement [2]. V4R has 93% sensitivity and 95% specificity for RVI [3].

## Materials and methods

This cross-sectional study was conducted at the Department of Cardiology, Quaid-e-Azam Medical College. A total of 1000 patients with acute inferior wall myocardial infarction (IWMI) were evaluated. Elevated ST-segment elevations in chest leads of ECG V3R and V4R were used to diagnose RVI. The duration for the collection of data was from 4th April 2019 to 29th March 2020. 

## Results

A total of 1000 patients of IWMI were included in the study. Diagnosis of inferior wall MI along with RVI was made on electrocardiogram (ECG) findings. All patients had ST-segment elevation in leads II, III, aVF in standard ECG, along with ST-segment depression in V2, V3, V4. On recording the right-sided ECG for those patients, there was ST elevation (minimum of 1 mm) in V3R, V4R, V5R.

Right ventricular infarct according to gender

Out of 1000 patients, 740 patients (74%) were male, and 260 patients (26%) were female. The number of patients having RV infarction among males was 260 (35% of males). The number of patients having RV infarction among females was 100 (38% of females). From the total number of RV infarct cases of IWMI, the incidence of RVI was higher in male patients compared with female patients (35.14% as compared to 38.46%). On calculations, the odds ratio was 0.87 (when males were compared to females) with a 95% confidence interval (CI) of 0.7 to 1.2 and P-value 0.3. This indicated that the finding was not statistically significant. 

Right ventricular infarct according to age group

The mean age of the patients was 52.05 years. Patients with ages between 30 and 40 years old, who suffered from IWMI, were 100. Out of these, 80 patients (80% of this age group) had RVI. Patients aged between 41 and 50 years were 140 in number, out of which 60 patients (43% of this age group) had RVI. Patients aged between 51 and 60 were 200 out of which 40 patients (20% of this age group) had RVI. Patients between the ages of 61 to 70 were 160 in number, out of which 40 patients (25% of this age group) had RVI. Patients between the ages of 71 and 80 years old 100 in number, out of which 40 patients (40% of this age group) had RVI.

Right ventricular infarct in patients having diabetes as a risk factor

The total number of patients with diabetes was 320, out of which 120 patients (37.5%) had an RVI. The total number of non-diabetic patients was 680, out of which 240 patients (35.2%) had RVI. From the total number of RVI cases of IWMI, the occurrence of RVI was higher in diabetic patients as compared to non-diabetic patients. But on calculations, the odds ratio was 1.1, with a 95% CI of 0.8 to 1.4, and a P-value of 0.5. This indicated that the finding was not statistically significant. 

Right ventricular infarct in patients with smoking as a risk factor

The total number of patients who were active smokers in this study was 540, out of which 300 patients (56% of smokers) had RVI. The total number of patients who were non-smokers was 460, out of which 220 patients (48% of non-smokers) had RVI. From the total number of RVI cases of IWMI, the occurrence of RV infarct was higher in non-smoker patients compared with patients who smoke. On calculations, the odds ratio was 1.4, with a 95% CI of 1.1 to 1.8 and a P-value <0.01. This indicated that the finding was statistically significant.

Right ventricular infarct in patients with hypertension as a risk factor

The total number of patients with hypertension as a comorbidity in this study was 560, out of which 240 (43% of hypertensive patients) had RVI. The total number of non-hypertensive patients was 440, out of which 120 patients (27 % of non-hypertensive patients) had RVI. The occurrence of RVI was higher in hypertensive patients as compared to non-hypertensive patients. On calculations, the odds ratio was 2.0, with a 95% CI of 1.5 to 2.6, and P-value <0.0001. This indicated that the finding was statistically significant.

## Discussion

In the present study, 1000 patients were studied to assess the incidence and clinical profile of RVI in acute IWMI patients. A total of 360 (36%) had right ventricular MI out of a total of 1000 IWMI patients. Khandait V et al. had found 45 (30%) out of 150 cases of IWMI to be having right ventricular involvement [4]. Ravikeerthy M et al. observed the incidence at 40% [5]. Memon AG et al. observed that out of 198 cases with IWMI, 96 (48.5%) cases had evidence of RVMI [6]. Iqbal A et al. found 16 (32%) cases of IWMI to be having right ventricular involvement [7]. Thus, the author’s findings concerning the incidence of RVMI in IWMI are mostly supportive of the available evidence. RVI is less common since the right ventricle is less susceptible to ischemia as oxygen demand is significantly lesser because of its smaller muscle mass, and coronary perfusion in the right ventricle occurs in both systole and diastole [2].

The mean age of the patients was 52.05 years. Most of the cases (42.8%) of RVI occurred in the age group of 41-50 years in the RVI group compared to non-RVI that was commonly (75%) seen in the age group of 61-70 years. In the present study, 74% were males. A similar male preponderance was reported in other studies [4-5]. Among the 740 male patients in our study, 260 (35%) had RVI, while only 100 of the 260 females (38%) had evidence of RVI. Our study showed that the male gender did not hold any statistically significant link to the incidence of right ventricular infarct in patients. This might be due to the low occurrence of certain other risk factors in this sample of women, like smoking. This finding contradicts the research by Obradovic S, et al. of the increased incidence of RVI in females [8]. Our sample of women was from a southeast culture where smoking is less common in the female gender [9].

It was observed in the present study that out of a total of 1000 participants, 540 (54%) were smokers, out of which 300 (56%) had RV infarction. The odds ratio was 1.1, showing statistical significance in our study and associating higher chances of RVI in IWMI if smoking is one of the risk factors. This is in line with the findings by Memon AG et al. and Iqbal A et al., where smoking is a significant risk factor for RVI in patients with IWMI [6-7].

Our study also showed that there could be an increased risk of RVI in IWMI if diabetes is one of the risk factors, having an odds ratio of 1.1, with a 95% confidence interval (CI) of 0.8 to 1.4 and P-value of 0.5. Although this is not statistically significant, there could be some clinical significance that needs to be explored further. Similar results were reported by Khandait V et al. and Ravikeerthy M et al., where diabetes was found in 45.3% and 24% cases, respectively, where their results were statistically significant [4-5]. Our statistical significance could be linked to the fact that we did not test for diabetes. Instead, the number of patients in our study who had diabetes were those who were already diagnosed. This is opposite to the study done by Khandait V et al., who tested for diabetes in all the patients, not only those who had a history of diabetes [4].

Our study also showed that there is an increased risk of RVI in IWMI if hypertension is one of the risk factors, having an odds ratio 2.0, with a 95% confidence interval (CI) of 1.5 to 2.6, and P-value <0.0001. Khandait V et al. had studied 150 cases, out of which 87 (58%) were hypertensive, which is in concordance with the observations made by us, while the findings of Ravikeerthy M et al. and Memon AG et al. do not correlate with the results of our study [4-6].

## Conclusions

RVI can accompany IWMI in almost one-third of the cases. It is essential to look for risk factors in chronic heart disease patients. This simple approach may facilitate early identification and risk stratification of patients who might have an increased incidence of RV infarct in IWMI. These risk factors include hypertension, diabetes mellitus, and smoking. Out of these three, the odds of RVI are highest in patients with hypertension. Hence, we conclude it is one of the stronger risk factors for RVI in IWMI patients. Timely control of said risk factors will result in reduced incidence and hence the complication associated with RVI.

This study has potential limitations which could be reflected in the results; these include:

(1) A cross-sectional design, which meant that it was not possible to determine the causal association between risk factors and RVI.

(2) The findings may be limited because of the inability to test all patients for diabetes (the data used for diabetic patients includes those patients who were already diagnosed) because of financial limitations.

The strengths of our study include:

(1) The sample was representative of the target population; therefore the external validity of the study is likely to be high.

(2) There was a satisfactory response rate for the assessment.

(3) A large sample size.
